# Tobacco Use and Second-Hand Smoke Exposure Among Athletes; Assessment by Urine Cotinine Level and Exhaled Carbon Monoxide: A Cross-Sectional Study

**DOI:** 10.3390/healthcare13020198

**Published:** 2025-01-20

**Authors:** Erdogan Asar, Yunus Emre Bulut, Nermin Dindar Badem, Aydan Orscelik, Cagri Emin Sahin, Gokhan Buyukluoglu, Ismail Kucuk, Tugba Kocahan, Toker Erguder

**Affiliations:** 1 Department of Medical Informatics, Gulhane Faculty of Medicine, University of Health Sciences, Ankara 06018, Türkiye; 2 Department of Public Health, Gulhane Faculty of Medicine, University of Health Sciences, Ankara 06018, Türkiye; yunusemre.bulut@sbu.edu.tr (Y.E.B.); drceminsahin@gmail.com (C.E.S.); ergudert@who.int (T.E.); 3 Department of Medical Biochemistry, Gulhane Faculty of Medicine, University of Health Sciences, Ankara 06018, Türkiye; ndindar06@yahoo.com; 4 Department of Sports Medicine, Gulhane Faculty of Medicine, University of Health Sciences, Ankara 06018, Türkiye; aydanozcan@yahoo.com (A.O.); gokhanbuyukluoglu@gmail.com (G.B.); kocahantu@gmail.com (T.K.); 5 Department of Institute Epidemiology, Istanbul Medipol University, Istanbul 34810, Türkiye; 6 Department of Sports Medicine, Ministry of Health Ankara Gülhane Training and Research Hospital, Ankara 06010, Türkiye; ismail.kuckk@gmail.com; 7 World Health Organization Turkiye Country Office, Ankara 06550, Türkiye

**Keywords:** athletes, tobacco and tobacco products use, smoke-free policy, second-hand smoke exposure, urine cotinine and carbon monoxide levels

## Abstract

**Background and Objectives:** Tobacco use and exposure to tobacco products remain a major public health challenge, even among athletes. This study aimed to evaluate tobacco use and second-hand smoke exposure in athletes through urinary cotinine levels and exhaled air carbon monoxide (CO) measurements. **Materials and Methods:** This cross-sectional study included licensed athletes who visited the sports medicine outpatient clinic of a tertiary hospital. Participants completed an electronic questionnaire on smoking habits and second-hand smoke exposure. Urine samples were analysed for cotinine levels, while CO levels were measured in exhaled breath. **Results:** A total of 421 athletes participated (57.8% male, median age: 18 years). Tobacco use was reported by 29.9% (*n* = 126) of participants. Median urinary cotinine levels were 22.9 ng/mL among daily tobacco users and 17.4 ng/mL among athletes exposed daily to second-hand smoke, with a statistically significant difference between exposure levels (*p* < 0.05). The optimal cut-off value for detecting second-hand smoke exposure was identified as 12.5 ng/mL. Median exhaled air CO levels were 5 ppm in daily tobacco users. **Conclusions:** This study demonstrates that tobacco use prevalence among athletes mirrors Türkiye’s national rates. Despite legal measures to curb tobacco use, direct use and second-hand smoke exposure remain pressing issues among athletes.

## 1. Introduction

The use of tobacco and tobacco products represents a significant public health concern on a global scale, with over 8 million deaths estimated to be attributed to its consumption annually [1]. The daily use of these products among the Turkish population aged 15 years and older increased from 28.0% in 2019 to 28.3% in 2022 [2]. The adverse effects of tobacco and tobacco products are numerous and diverse. In order to combat these effects, the World Health Organization (WHO) has developed a series of policies under the WHO Framework Convention on Tobacco Control (WHO FCTC). Türkiye signed and ratified the WHO FCTC in 2004. Examples of these policies include the implementation of a smoking ban in enclosed spaces, the display of warnings on cigarette packs, the imposition of increased taxes on tobacco products, and the dissemination of awareness-raising campaigns. Nevertheless, further measures are required to safeguard the younger demographic and athletes [1,2].

The use of tobacco and tobacco products among athletes is becoming an increasing concern due to the detrimental effects that these substances have on both health and performance. For instance, a study conducted in Qatar revealed that 27.7% of professional athletes were found to be using these products [3]. In other studies, a reduction in lung function has been observed in athletes who smoked in comparison to non-smokers [4]. The targeting of young athletes by tobacco companies increases the risk of exposure to second-hand smoke in sports environments. This has prompted concerns regarding the exposure of non-smokers to second-hand smoke in sports environments, leading health organisations such as the WHO to emphasise the protection of non-smokers from these harmful effects [5,6].

Tobacco use is a major risk factor for respiratory and cardiovascular diseases, as it increases heart rate and blood pressure and reduces blood oxygen levels. While nicotine can temporarily enhance alertness, its long-term use is harmful and addictive. Cotinine, a metabolite of nicotine with a 16–20 h half-life, is used to monitor nicotine exposure and assess the health risks of tobacco use [7,8].

Second-hand smoke exposure has also been demonstrated to have a significant impact on human health. The evidence indicates that second-hand smoke exposure also has acute and short-term effects on the cardiovascular and respiratory systems. For instance, second-hand smoke exposure for a mere one hour at levels that are typical for bars and restaurants can have a deleterious effect on cardiorespiratory and immune responses to maximal physical exertion in healthy non-smokers for at least three hours after exposure. In non-smokers of second-hand smoke exposure, the risk of developing coronary heart disease is increased by 25–30%, while the risk of stroke rises by 20–30%. Even brief second-hand smoke exposure may elevate the risk of atherosclerosis and myocardial infarction [9,10,11].

The objective of this study was to evaluate tobacco and tobacco product use, as well as second-hand smoke exposure, in licensed athletes through the analysis of urinary cotinine and inhaled air CO levels. The results of this study will facilitate a comprehensive understanding of the prevalence and motivations behind tobacco use among athletes and will contribute to the development of strategies by which to reduce tobacco use in this population.

## 2. Materials and Methods

The study population comprised licenced athletes (with the license certificate given to athletes by the authorized institution, athletes can participate in training and competitions in the sports branch they play) between the ages of 15 and 40 and who had applied to the Sports Medicine Outpatient Clinic of the University of Health Sciences Gülhane Training and Research Hospital in Ankara between 1 November 2023 and 31 January 2024 for any reason.

Ethical approval was obtained from the Health Sciences University Gülhane Training and Research Hospital Clinical Research Ethics Committee (Decision date: 27/09/2023, decision no: 2023/195). The study was conducted in accordance with the procedures of the Declaration of Helsinki.

The power analysis of the study was conducted using the G*Power software (version 3.1.9.7). The test was powered at 95%, the effect size was 0.20 (Cohen f = 0.20), and the significance level was set at 5%. The power analysis yielded a total sample size of 390. A 15% contingency was added to this figure to allow for potential exclusions and other factors, resulting in a final sample size of 449.

### 2.1. Study Design

In the study, an electronically administered questionnaire was prepared based on the literature review. Before administering this questionnaire to the participants, a pre-test study was conducted and the questionnaire form was finalized. This questionnaire was designed in a way that would not allow participants to skip the questions without answering them and would therefore not have any missing data (the number of participants was determined by taking these and similar problems into consideration). The questionnaire, which was administered under the supervision of the researchers, included questions about the sociodemographic characteristics of the participants, tobacco and tobacco product use, the type of tobacco and tobacco products used, the duration of use, the level of addiction, and exposure to second-hand smoke from these products [12,13]. The participants were classified according to their level of addiction, which was determined to be either high and moderate, low to moderate and low [14].

Furthermore, the researchers conducted a series of anthropometric measurements to ascertain the height, body weight, and waist circumference of the athletes. Subsequently, CO was quantified in the exhaled air of the participants using an EC50 Smokerlyser device (Bedfont Instruments). A disposable mouthpiece was utilised for each participant during the measurement process.

Urine samples with a minimum volume of 10 mL were collected from the athletes who completed the measurements and placed into a sterile urine container for the determination of cotinine levels. The urine samples were subjected to centrifugation in a refrigerated centrifuge at 2000 rpm for 10 min, frozen within 30 min, stored at −80 °C, and brought to room temperature three hours prior to the commencement of the study. Cotinine concentrations in urine were determined using a commercially available ELISA kit (BT-LAB, Cat. No. E2043Hu) in accordance with the manufacturer’s instructions. The kit is capable of measuring concentrations between 0.5 ng/mL and 80 ng/mL with a sensitivity of 0.019 ng/mL, and the intra-assay and inter-assay coefficient of variation values are less than 8% and less than 10%, respectively. The kit is an enzyme-linked immunosorbent assay (ELISA) measurement kit. The plates included in the kit are pre-coated with an antibody specific to human cotinine. During the study, the urine samples were added to the wells, whereupon the cotinine present in the samples was bound to the antibodies that had been pre-coated on the wells. Subsequently, biotinylated human cotinine antibody was added and bound to the cotinine present in the sample. Streptavidin-HRP was then added and bound to the biotinylated cotinine antibody. After incubation, unbound streptavidin-HRP was removed by a washing step. Finally, substrate solution was added and colour developed in proportion to the amount of cotinine in the samples. The addition of acidic stop solution terminated the reaction and absorbance was measured at 450 nm. Pipetting was performed manually, and incubations were carried out at 37 °C. The study was conducted with a Biotek ELx50 ELISA washing device, a Biotek ELx800 ELISA reading device and the Alisei evaluation programme.

### 2.2. Patient and Public Involvement

Licenced athletes were included in this study. During the study’s implementation period, participants presenting to the Sports Medicine Outpatient Clinic were asked whether they were licensed athletes or not when they first arrived. Licensed athletes were asked whether they were between the ages of 15 and 40. Then, the athletes were informed about the subject of the study and the procedures to be performed and invited to work voluntarily. If the athlete accepted to participate in the study and was 18 years of age or older they were asked to read and sign the informed consent form approved by the ethics committee, which contained information about the content and procedure of the study; if the athlete was under 18 years of age, the athlete and his/her legal representative (mother, father etc.) were asked to read and sign the form. After this stage, the researchers provided comprehensive information to the athletes about the study and its processes. Athletes who did not have a license, were outside the age range of 15–40, and did not want to participate in the study, were not included in the study. In addition, those who completed the questionnaire but did not provide a urine sample or did not participate in the CO measurement application, and those who declared their participation but did not fill out the questionnaire completely were not included in the study. With the pre-test study, the opinions and suggestions of the athletes about the survey form were also received and evaluated. Electronic link addresses regarding the results of the study were to be shared with the athletes via their e-mail addresses or mobile phones.

### 2.3. Statistical Analysis

In group comparisons, Kruskal–Wallis analysis of variance (Kruskal–Wallis test) was performed for numerical data with a significance level of 5%. When there was a difference between the groups, Dwass–Steel–Critchlow–Fligner pairwise comparison test was used as post-hoc test. Comparisons of groups with categorical data were made with the chi-square test. The Shapiro–Wilk test was used to test the normality of the data. Determination of the best cut-off point was performed by receiver operating characteristic (ROC) analysis, a method based on sensitivity and specificity. Statistical analyses were performed using Jamovi software (version 2.3.28) and SPSS software (IBM SPSS for Windows, version 20.0).

## 3. Results

Of the 449 licensed athletes invited, 421 (93.8%) agreed to participate in the study. Among these athletes, 42.3% (*n* = 178) were female and 57.7% (*n* = 243) were male. The descriptive characteristics of the participants are presented in Table 1, and the frequency of tobacco and tobacco product use, as well as second-hand smoke exposure, are detailed in Table 2. It was determined that 29.9% (*n* = 126) of the participants were using tobacco and tobacco products. The prevalence of tobacco and tobacco product use was significantly higher among male athletes (61.1%) compared with their female counterparts (38.9%). The rates of those who used tobacco and tobacco products every day and those who never used tobacco and tobacco products were found to be statistically significant according to gender (*p* < 0.05), whereas no significant difference was found in occasional users (*p* > 0.05). Among the 295 athletes who did not use tobacco and tobacco products, 86.8% (*n* = 256) reported that they were passively exposed to tobacco smoke daily or at least once a week.

With regard to the educational status of the athletes, 28.9% (*n* = 122) were at the pre-high school level, 47.3% (*n* = 199) were in high school and 23.8% (*n* = 100) were in post-secondary education. A statistically significant difference was observed between the rates of tobacco and tobacco product users and non-users among athletes with pre-high school and high school education (*p* < 0.05). In athletes with post-secondary education, no statistically significant difference was observed between the rates of those who used and did not use these products (*p* > 0.05) (Table 3).

Urinary cotinine and carbon monoxide (CO) values according to the addiction levels of athletes using tobacco and tobacco products are given in Table 4. Accordingly, 6.4% of the athletes who used tobacco and tobacco products were found to exhibit moderate-to-high dependence, while 93.6% demonstrated minimal dependence.

Urinary cotinine evaluation results of athletes according to tobacco and tobacco products use and frequency of exposure to smoke of these products are given in Table 5. The differences between the medians, as determined by frequency of use, was statistically significant (*p* < 0.05). The post-hoc test demonstrated that this discrepancy was attributable to all pairwise comparisons (*p* < 0.05). The differences between the medians, as determined by frequency of exposure to smoke of these products, was statistically significant (*p* < 0.05). Post-hoc testing showed that this significant difference was due to all pairwise group comparisons (*p* < 0.05).

**Figure 1:**
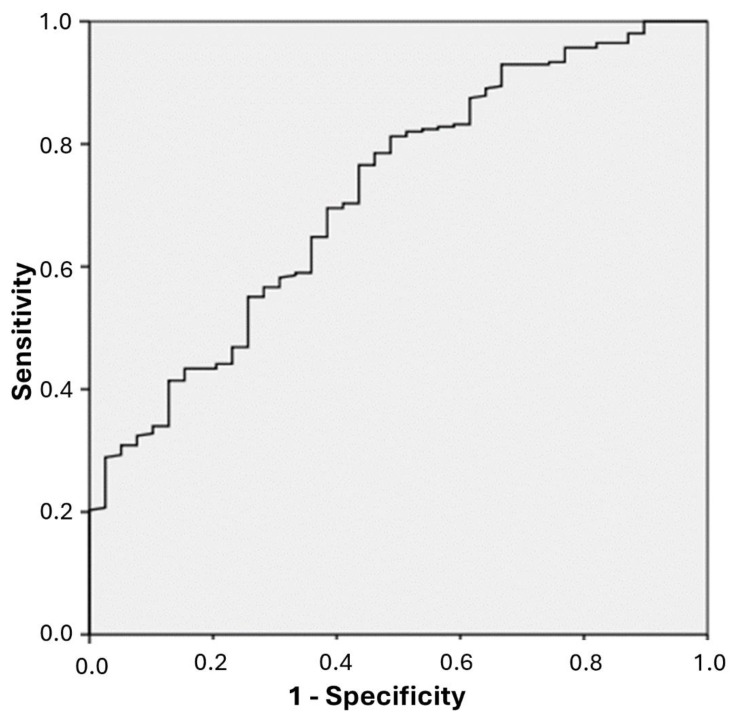
ROC curve for data regarding urinary cotinine in athletes exposed to tobacco and tobacco product smoke.

The ROC curve was used to determine the second-hand smoke exposure limit for athletes to tobacco and tobacco product smoke. The cut-off value for cotinine was set at 12.5 ng/mL using the Youden index. Any value above this was considered to be the second-hand smoke exposure limit. The area under the ROC curve was calculated to be 0.72. This area under the ROC curve was statistically significant (*p* < 0.05) and showed a moderate level of discrimination (Figure 1).

The median levels of exhaled CO of athletes who used tobacco and tobacco products and reported passive exposure to these products are given in Table 6. The differences between the medians, as determined by frequency of use of tobacco and tobacco product users, was found to be statistically significant (*p* < 0.05). The post-hoc test showed that this difference was due to the difference between the “daily users” and “non-users,” and “occasional users” and “non-users” groups (*p* < 0.05). The difference between the medians of the daily user and occasional user groups was not statistically significant (*p* > 0.05).

## 4. Discussion

Despite a decline in the prevalence of tobacco use among adults in Türkiye from 31.2% (16 million) in 2008 to 27.1% (14.8 million) in 2012, the proportion of adults who continue to use tobacco has remained above 25% [15]. The prevalence of daily tobacco use among individuals aged 15 years and above in Türkiye increased from 28.0% in 2019 to 28.3% in 2022 [16]. The prevalence of tobacco and tobacco product use among athletes in this study was 29.9%, which is comparable to the national average for Türkiye. A review of the literature reveals that elite/professional athletes are reported to have a significantly lower prevalence of smoking (0.8–15%) than the control group (17–28%) and the worldwide population (25%) [17,18,19]. Nevertheless, there are studies indicating that tobacco use is prevalent among athletes, particularly in team sports, and that its prevalence is on the rise [18,20]. A study conducted in Finland revealed a correlation between the use of smokeless tobacco and intensive physical activity among adolescents and young adults. The prevalence of smokeless tobacco use was found to be higher among those who engaged in intense physical activity, with an observed increase over time [20]. In another study conducted in Switzerland, it was observed that the prevalence of smokeless tobacco use, especially snuff, increased in parallel with the intensity of exercise [21]. In another study, it is reported that the prevalence of daily cigarette smoking among 446 elite athletes in Finland was one in seven, when compared with the general population. Daily snuff use was also found to be five times higher than in controls [17]. A study found that young people engaged in high-intensity sports were less likely to smoke than those engaged in low-intensity sports [22]. In a survey of 546 athletes in Ireland, it is reported that 16% of the participants were current smokers, 10% of all participants were exposed to second-hand smoke for more than one hour per day, and 2% of all participants were current electronic cigarette users [23]. The prevalence of daily tobacco and tobacco product use in Türkiye was found to be 41.3% among men and 15.5% among women in 2022 [24]. In 2020, it was reported that, globally, approximately 38% of men aged 15 years and older were tobacco users [25]. In this study, the prevalence of daily cigarette smoking among male athletes was 21.8%, which is below the national average in Türkiye. In a study conducted on 108 male professional athletes in Qatar, the prevalence of tobacco use (27.7%) was higher than the prevalence in the general Qatari male population (21.1%) [3]. Although it is reported in the literature that having a high level of education decreases the probability of using tobacco products [20], it was observed in this study that the use of tobacco and tobacco products increases with the increase in the level of education. The findings in our study suggest that the level of education increases as the age increases and that the use of tobacco and tobacco products increases in parallel. This situation explains the increase in the rate of tobacco and tobacco product use in athletes as they age, similar to the general population in Turkey [26]. A number of studies have indicated that the consumption of smokeless tobacco products is more prevalent among athletes [17,20,21,22]. However, the findings of this study indicate that athletes exhibited a greater proclivity for smoking tobacco and tobacco products (62.1%), with hookah (19.3%) and electronic cigarettes (18.6%) representing the second and third most preferred alternatives, respectively.

Among the athletes who participated in the study, 6.4% were found to exhibit moderate to high levels of addiction, while 93.6% demonstrated minimal to mild addiction. In a study examining the tobacco use habits of adolescent athletes, it was found that 8% of the athletes showed moderate-to-high levels of addiction symptoms, while the remaining 92% exhibited low levels of addiction. In other words, as evidenced in the literature, the prevalence of a low level of addiction is considerably higher, yet the frequency of use has increased [23]. The composition of cigarette smoke is highly complex, comprising over 7000 distinct chemical compounds. Among these, nicotine is widely acknowledged as a pivotal contributor to tobacco addiction. Nicotine exerts a psychoactive effect by increasing the transmission of the neurotransmitter acetylcholine and the release of the neurotransmitter dopamine in the brain. The act of smoking is undertaken by daily smokers for the purpose of maintaining optimal levels of the psychoactive substance nicotine within the brain, as well as to achieve a state of arousal. Furthermore, there is a correlation between smoking and sleep disturbances, both during regular intake and after smoking cessation [19,27]. The use of nicotine, particularly in its smokeless forms, is prevalent among athletes, with the assertion that it mitigates anxiety, enhances concentration and agility, and facilitates weight management [28]. It has been demonstrated that the use of tobacco products has an adverse effect on an individual’s aerobic capacity and muscle performance. Additionally, it has been observed that athletes with a nicotine addiction can enhance their athletic performance and physical function by ingesting nicotine 15 to 30 min prior to competitions. However, the literature also indicates that abstaining from nicotine for a period of at least three months following the recovery from addiction can lead to a notable enhancement in athletic performance [29,30,31,32]. In contrast, short-term nicotine abstinence (i.e., ≤24 h) has been demonstrated to markedly impair the performance and physical cognition of exercisers. Consequently, the World Anti-Doping Agency (WADA) included nicotine in its monitoring programme in 2012 due to its psychoactive effects, and it is also included in the monitoring programme for 2024, although it is not recognized as a banned substance [33]. A study has demonstrated that electronic and tobacco cigarettes have comparable effects on serum cotinine levels following both active and passive smoking. Electronic cigarettes have been demonstrated to elicit nicotinergic effects comparable to those observed in tobacco cigarettes [34].

In the present study, 86.8% of athletes who did not use tobacco and tobacco products were passively exposed to these product smoke. Of these, 47.1% were exposed to the smoke of these products on a daily basis, while 39.7% were exposed at least once a week. A large-scale global study investigating second-hand smoke exposure among adolescents, conducted between 2010 and 2018, revealed that, while the prevalence of exposure at home declined, the prevalence of exposure in public places increased or remained unchanged in the majority of countries between 1999 and 2018 [35]. The issue of second-hand smoke exposure among young people represents a significant public health concern on a global scale. It is estimated that second-hand smoke exposure causes more than 1.2 million premature deaths and serious cardiovascular and respiratory diseases annually. It is not possible to determine a safe level of second-hand smoke exposure. The implementation of smoke-free legislation serves to safeguard the health of non-smokers, while also enjoying considerable popularity due to its role in encouraging smokers to quit [36]. A cut-off value of 12.5 ng/mL for cotinine was identified as the limit of second-hand smoke exposure to tobacco and tobacco product smoke in the present study. The median cotinine value in urine was 22.9 ng/mL in individuals who used tobacco and tobacco products on a daily basis, 18.00 ng/mL in those who used tobacco and tobacco products on an occasional basis, and 15.9 ng/mL in athletes who reported never using tobacco and tobacco products. However, the urinary cotinine value of the athletes remained above the cut-off value, indicating that both users and non-users were exposed to second-hand tobacco smoke. Despite the successful enactment of legal regulations aimed at combating tobacco and tobacco products in Türkiye, the prevalence of tobacco and tobacco product use remains considerable. While sports fields have been designated as smoke-free zones, the exposure of athletes to second-hand tobacco smoke is a notable concern. In this study, the median cotinine value of athletes who reported being exposed to second-hand tobacco smoke every day was found to be 17.4 ng/mL; 15.6 ng/mL for those exposed at least once a week; and 11.5 ng/mL for those who reported rarely being exposed. The urine cotinine value of athletes who reported rarely being exposed to second-hand tobacco and tobacco product smoke was found to be below the cut-off value of 12.5 ng/mL. The urine cotinine level was found to be above the calculated cut-off value in athletes who declared that they smoked and in athletes who reported being exposed to second-hand smoke. This shows that tobacco and tobacco product use and second-hand exposure in sports areas are serious among athletes. The fact that a population with a healthy life expectancy has a similarly high level of exposure to the general population is concerning. As can be understood from these results, the athletes’ statements and urine cotinine results support each other. This shows that the questionnaire applied in the study was understood and answered correctly by the athletes, and that the urine samples taken from the athletes were successful in detecting second-hand smoke exposure in athletes.

In this study, the median CO values in the exhaled air of the athletes were 5 ppm in those who used tobacco and tobacco products every day, 3 ppm in those who used them occasionally, and 1 ppm in athletes who reported never using them. The median CO values were found to be 1 ppm in athletes who were exposed to second-hand tobacco smoke and tobacco products and those who were rarely exposed. Despite the lack of discernible difference in median CO values according to the frequency of exposure to tobacco and tobacco product smoke, a statistically significant difference was observed between the median CO values in the exhaled air of those who used and did not use tobacco and tobacco products (*p* < 0.05). In general, without potential air pollution, exhaled CO concentrations are expected to be in the range of 1–4 ppm in non-smokers and 2–18 ppm in smokers [37]. In a study, the mean basal exhaled CO concentration was found to be 6.9 ± 4.9 ppm in smokers and 1.9 ± 0.5 ppm in non-smokers. The mean exhaled CO half-life for smokers was reported to be 4.6 h [38].

Areas designated for sporting activities are to be maintained as smoke-free environments. In accordance with standard practice, the use of tobacco and tobacco products is prohibited in these areas. In addition to sports areas, indoor areas are also designated as smoke-free airspaces. The rate of second-hand smoke exposure was 86.8% in this study. This indicates that tobacco and tobacco products are being used in these areas. In Türkiye, the pertinent legislation, adopted in 2008, prohibits the consumption of tobacco products in indoor areas. However, it is perceived that the regulatory controls are insufficient and that there are some deficiencies in their implementation [39]. The WHO FCTC recognizes the significant harm caused by tobacco use and the critical need to prevent it. In 2008, the WHO identified six evidence-based tobacco control measures that are most effective in reducing tobacco use. These measures, known as “MPOWER,” correspond with one or more of the demand reduction provisions in the WHO FCTC. Although the entire population in Türkiye is currently protected at the highest level by MPOWER measures, inspections in public areas are considered inadequate [2,39].

One of the strengths of our study is that urine cotinine levels were examined in all athletes in order to verify tobacco and tobacco product use and exposure information. The other strength of this study is that the participants in this study were part of a young population (median age 18.0 years), that they were licensed athletes, and that both women and men were included (42.3% women, 57.7% men).

### Limitations

In this study, data from athletes were collected using a self-reported questionnaire. Some athletes may not have given correct answers to questions about tobacco and tobacco product use, which is one of the limitations of the study. Another limitation of this study is that participants were not asked about the time of their last tobacco or tobacco product use or their exposure to second-hand smoke before CO measurement.

## 5. Conclusions

This study examines the prevalence of tobacco and tobacco product use, as well as the extent of second-hand smoke exposure among athletes in Türkiye. The findings provide compelling evidence that tobacco use and second-hand smoke exposure should be reduced among athletes. The findings of the study, which were corroborated by urine cotinine levels, indicate that the prevalence of tobacco use and exposure to second-hand smoke among athletes in Türkiye is comparable to that observed in the general Turkish population. Furthermore, the prevalence of second-hand smoke exposure among athletes is a significant concern. It is therefore recommended that sports fields and indoor spaces be subjected to more rigorous inspection, and that all athletes and their support personnel be provided with comprehensive training on the adverse effects of tobacco and tobacco products and the benefits of quitting. The findings of our study must be taken seriously by those involved in sports, and measures must be implemented to protect the health of athletes. In the future, when planning more comprehensive studies, examining tobacco use and second-hand smoke exposure among athletes, together with the effects of oxidative stress biomarkers, cardiovascular health measurements and athlete performance, etc. may be considered. In addition, it would be very useful to include issues such as athletes’ success, age, type of sport, level of education, etc. in such future studies.

## Figures and Tables

**Table 1:** The descriptive characteristics of the participants.

Variables	Mean	Median	Standard Deviation	Min	Max
Age (year)	19.5	18.0	5.5	15.0	40.0
Body weight (kg)	66.7	64.0	13.9	40.0	125.0
Height (cm)	172.0	172.0	9.4	135.0	200.0
Waist circumference (cm)	77.0	75.0	12.6	55.0	176,0
Urine cotinin value (ng/mL)	18.0	17.0	7.1	0.3	54.1
CO in exhaled air (ppm)	2.6	1.0	3.3	0.0	22.0
Sports age (year)	6.2	5.0	5.4	1.0	30.0

*n*: number of sampling units: 421; CO: carbon monoxide.

**Table 2:** Distribution of tobacco and tobacco products used by athletes and the frequency of use and exposure by gender.

				Gender					
				Female		Male			
		*n*	% ^a^	*n*	% ^b^	*n*	% ^b^	Χ^2^	*p* Value
Tobacco/ tobacco products (*)	Cigarettes, rolled cigarettes, cigars, pipes	177	62.1	72	40.7	105	59.3	6.2	<0.05
	Hookah	55	19.3	21	38.2	34	61.8	3.1	>0.05
	Electronic cigarette	53	18.6	21	39.6	32	60.4	2.3	>0.05
Frequency of use	Daily	83	19.7	30	36.1	53	63.9	6.4	<0.05
	Occasional	43	10.2	19	44.2	24	55.8	0.6	>0.05
	None	295	70.1	129	43.7	166	56.3	4.6	<0.05
Exposure frequency	Daily	139	47.1	62	44.6	77	55.4	1.6	>0.05
	At least once a week	117	39.7	46	39.3	71	60.7	5.3	<0.05
	Rarely	39	13.2	21	53.8	18	46.2	0.2	>0.05

*n*: number of sampling units; χ^2^: χ^2^ test statistic; a: column percentage is used; b: row percentage is used. (*): Since a person can use more than one tobacco and tobacco products, there are differences between the number of smokers in this table and the number of types of tobacco and tobacco product use.

**Table 3:** Distribution of athletes using tobacco and tobacco products by their educational status.

	Usage Status of Tobacco and Its Products					
	User		Non-User			
Educational Status	*n*	%	*n*	%	χ^2^	*p* Value
Pre-high school	18	14.8	104	85.2	60.6	<0.05
High school	52	26.1	147	73.9	45.4	<0.05
Post-secondary education	56	56.0	44	44.0	1.44	>0.05

*n*: Number of sampling units.

**Table 4:** Urinary cotinine and carbon monoxide (CO) values according to the addiction levels of athletes using tobacco and tobacco products.

Variables	Addiction Level	*n*	%	Mean	Median	Standard Deviation	Min	Max	KW	*p* Value
Cotinine (ng/mL)	Low	94	74.6	22.8	20.7	8.1	12.2	54.1	1.9	>0.05
	Low to moderate	24	19.0	23.6	21.9	7.4	14.0	36.4		
	High and moderate	8	6.4	26.5	25.4	9.5	12.6	46.1		
CO (ppm)	Low	94	74.6	4.3	3.0	3.9	0.0	20.0	2.9	
	Low to moderate	24	19.0	4.8	4.0	4.3	0.0	14.0		>0.05
	High and moderate	8	6.4	7.8	7.0	6.5	1.0	22.0		

*n*: number of sampling units; KW: Kruskal–Wallis test statistic.

**Table 5:** Urinary cotinine evaluation results of athletes according to tobacco and tobacco product use and frequency of exposure to smoke of these products.

				Cotinine (ng/mL)						
		*n*	%	Mean	Median	Standard Deviation	Min	Max	KW	*p* Value
Frequency of use	Daily	83	19.7	24.6	22.9 ^a^	8.1	12.6	54.1	82.0	<0.05
	Occasional	43	10.2	20.5	18.0 ^b^	7.5	12.2	49.3		
	None	295	70.1	15.8	15.9 ^c^	5.4	3.3	30.9		
Exposure frequency	Daily	139	47,1	17.3	17.4 ^a^	5.3	4.2	30.9	26.1	<0.05
	At least once a week	117	39.7	15.3	15.6 ^b^	4.7	1.5	22.8		
	Rarely	39	13.2	12.0	11.5 ^c^	5.4	0.3	20.8		

*n*: number of sampling units; KW: Kruskal–Wallis test statistic; ^a,b,c^: the difference between exposure frequencies whose medians are shown with different letters is significant (*p* < 0.05).

**Table 6:** Evaluation of carbon monoxide (CO) inhalation based on athletes’ use of tobacco and tobacco products and their exposure to harmful smoke.

				CO (ppm)						
		*n*	%	Mean	Median	Standard Deviation	Min	Max	KW	*p* Value
Frequency of use	Daily	83	19.7	5.1	5.0 ^a^	4.6	0.0	22.0	62.1	<0.05
	Occasional	43	10.2	3.6	3.0 ^a^	3.2	0.0	15.0		
	None	295	70.1	1.7	1.0 ^b^	2.4	0.0	22.0		
Exposure frequency	Daily	139	47.1	2.1	1.0	3.1	0.0	22.0	^(1)^	
	At least once a week	117	39.7	1.4	1.0	1.2	0.0	7.0		
	Rarely	39	13.2	1.5	1.0	1.2	0.0	5.0		

*n*: number of sampling units; KW: Kruskal–Wallis test statistic; ^(1)^: no statistical test was performed as the median values were the same; ^a,b^: the difference between exposure frequencies whose medians are shown with different letters is significant (*p* < 0.05).

## Data Availability

Dataset available on request from the authors. The raw data supporting the conclusions of this article will be made available by the authors on request.
